# Macroscopic pulmonary fat embolism following cemented hemiarthroplasty for pathological femoral fracture: A case report with dual-energy CT spectral analysis

**DOI:** 10.1016/j.radcr.2026.10.034

**Published:** 2026-11-05

**Authors:** Hussayn Chettab, Sofyan Jankowski, Guillaume Fahrni, David C. Rotzinger

**Affiliations:** Department of Diagnostic and Interventional Radiology, Lausanne University Hospital (CHUV) and University of Lausanne (UNIL), Lausanne, Switzerland

**Keywords:** Pulmonary fat embolism, Cemented hemiarthroplasty, CT pulmonary angiography, Dual-energy CT, Nonsmall cell lung cancer, Spectral analysis

## Abstract

Macroscopic pulmonary fat embolism is an uncommon complication of cemented arthroplasty, and its occurrence after fixation of a pathological femoral fracture is rarely documented. We report the case of a 73-year-old woman with metastatic nonsmall cell lung cancer who developed severe hemodynamic instability during cement pressurization for hemiarthroplasty of a pathological right proximal femoral lesion, consistent with bone cement implantation syndrome but not fulfilling the criteria for fat embolism syndrome, and who recovered with supportive care. Dual-energy CT pulmonary angiography showed a 30 × 15 mm filling defect in the right main pulmonary artery and smaller left lower lobe segmental defects, with a mean attenuation of −86.6 HU within the main defect, CT numbers becoming progressively more negative at lower photon energies in contrast to a paraspinal muscle reference, and persistence of the defect on virtual noncontrast images. This case illustrates that attenuation measurement within any postoperative intraluminal filling defect is a simple and potentially decisive step, and that dual-energy spectral analysis may provide complementary support for the fatty nature of an embolus.

## Introduction

Fat embolism syndrome (FES) is a well-recognized but underdiagnosed complication of long bone fractures and orthopedic procedures. Its reported incidence is highly variable depending on diagnostic criteria and study design, ranging from less than 1% in retrospective clinical series to 11%-19% in prospective studies [1]. A systematic review reported a decreasing trend over recent decades, with incidence ranging from 0.8% to 23% across studies [2]. In the same review, fat embolism syndrome was reported in 3.3% of pathological fractures, compared with 2.9% of unilateral high-energy fractures and 4.6% of bilateral high-energy fractures [2]. The pathophysiology involves the release of fat globules from the bone marrow into the venous circulation, leading to mechanical obstruction, biochemical injury to the pulmonary vasculature and a systemic inflammatory response [1]. While fat embolism most commonly occurs in the context of traumatic fractures in young patients, fat embolization following cemented arthroplasty for pathological lesions due to bone metastases represents a distinct and particularly challenging clinical scenario.

A closely related phenomenon, bone cement implantation syndrome (BCIS), is defined as hypoxia, hypotension or unexpected loss of consciousness occurring around the time of cementation or prosthesis insertion during cemented arthroplasty [3]. The underlying mechanism involves an increase in intramedullary pressure during cement pressurization that forces fat and marrow content into the venous circulation [4]. Bone metastases and pathological fractures are recognized risk factors for BCIS, and patients with femoral metastasis undergoing cemented arthroplasty are considered at particularly high risk of developing severe forms of the syndrome [5].

CT pulmonary angiography (CTPA) plays a central diagnostic role in this setting, not only by detecting intraluminal filling defects but also by quantifying their CT number. Fat emboli demonstrate negative attenuation values on CTPA, typically ranging from −50 to −150 HU [6], allowing differentiation from acute thrombotic emboli which typically demonstrate a mean attenuation of ca. +33 HU and chronic thrombotic emboli around +87 HU [7]. Moreover, spectral CT is increasingly used in clinical practice, providing refined material characterization that has been proven helpful in animal models [8]. However, direct spectral characterization of macroscopic fat emboli in humans has rarely been reported.

We present the case of a 73-year-old woman with metastatic NSCLC who developed macroscopic pulmonary fat embolism immediately following cemented hemiarthroplasty for a pathological proximal femoral lesion, diagnosed on dual-energy CTPA including spectral analysis.

## Case presentation

A 73-year-old woman with a known history of metastatic nonsmall cell lung cancer (NSCLC, stage T3N3M1c IVb) was referred to our institution from a regional hospital for surgical management of an impending pathological fracture of the right proximal femur. Her oncological treatment consisted of a palliative chemo-immunotherapy regimen combining carboplatin, paclitaxel, and pembrolizumab. Her medical history was notable for type 2 diabetes mellitus, arterial hypertension, dyslipidemia, chronic kidney disease (KDIGO stage G4 on diabetic and hypertensive nephropathy), and paranoid schizophrenia.

She presented with right hip pain and functional impairment. Pelvic radiographs and CT demonstrated an osteolytic metastatic lesion of the right femoral head with extension to the lesser trochanter and cortical lysis, consistent with an impending pathological fracture (Fig. 1D). Following multidisciplinary discussion, cemented right hemiarthroplasty with proximal femoral reconstruction using a long-stem prosthesis and trochanteric plate fixation was performed a week later.

**Fig. 1:**
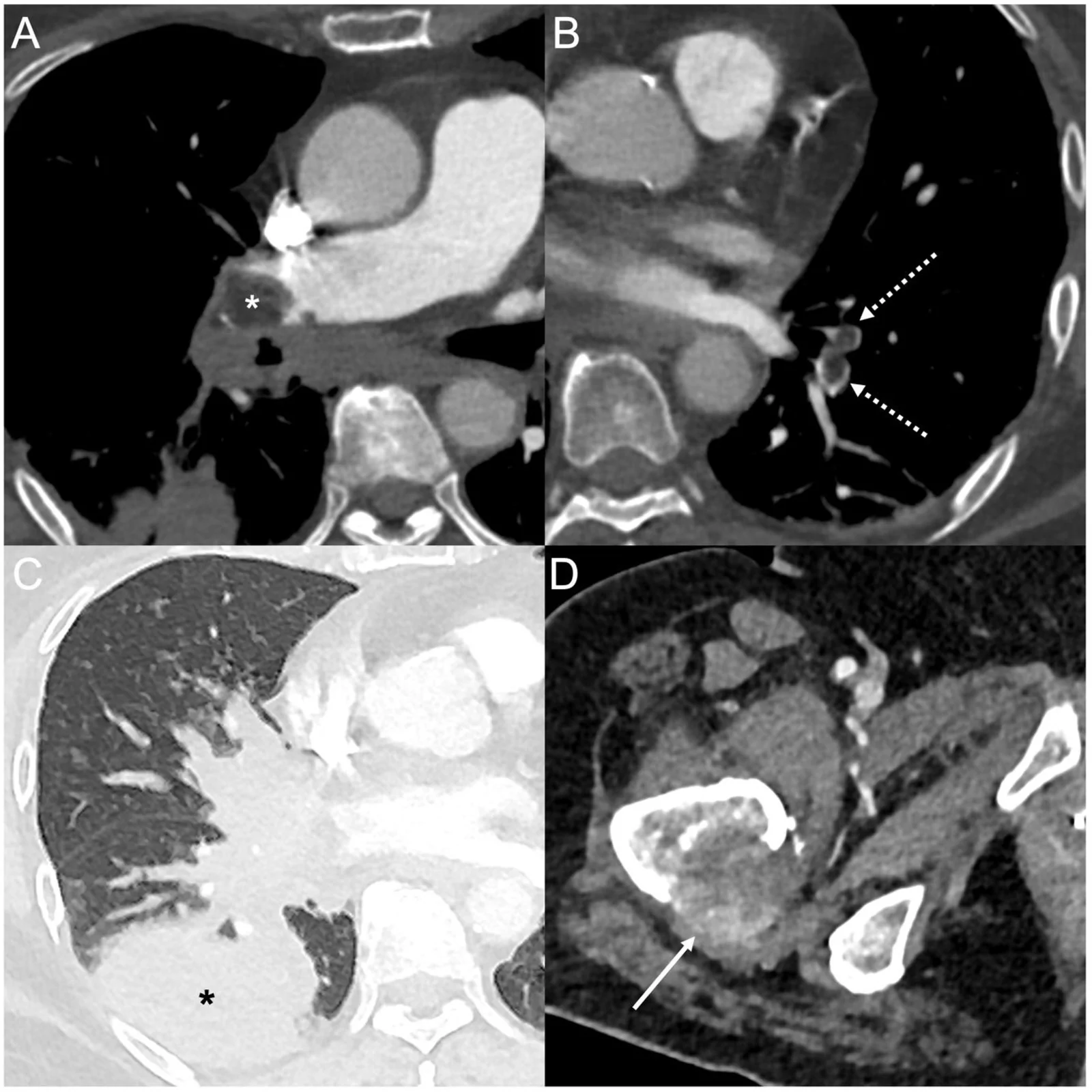
CT imaging features of pulmonary fat embolism and underlying oncological disease in a 73-year-old woman with metastatic nonsmall cell lung cancer. (A) Axial CT pulmonary angiography (mediastinal window) demonstrating a large intraluminal filling defect in the right main pulmonary artery (white asterisk), with predominantly low attenuation values (mean CT number −86.6 HU at 55 keV) consistent with macroscopic fat embolism. (B) Axial CT pulmonary angiography (mediastinal window) showing additional filling defects in segmental branches of the left lower lobe (white dashed arrows). (C) Axial CT of the thorax (parenchymal window) showing the primary right lower lobe pulmonary mass (black asterisk). (D) Axial CT of the pelvis (soft tissue window) demonstrating an osteolytic metastatic lesion of the right femoral head (white solid arrow) with cortical destruction, consistent with an impending pathological fracture, subsequently treated by cemented hemiarthroplasty.

During cement pressurization, the patient developed sudden and severe hemodynamic instability requiring high-dose vasopressor support. Intraoperative transesophageal echocardiography (TEE) demonstrated a suspected intraluminal filling defect within the right pulmonary artery, raising immediate concern for massive pulmonary embolism. The patient was kept intubated and transferred to the intensive care unit (ICU).

CT pulmonary angiography was performed on a dual-energy CT system (Revolution Apex, GE HealthCare) using a gemstone spectral imaging protocol with rapid kVp switching between 80 and 140 kVp. Acquisition parameters were as follows: helical mode, tube current 250 mA, rotation time 0.5 s, detector configuration 128 × 0.625 mm (total collimation 80 mm), pitch 1.375, table speed 220 mm/s, and a scan length of 280 mm for a total acquisition time of 1.66 s. Images were reconstructed at a slice thickness and interval of 1.25 mm with a standard soft-tissue kernel and deep learning image reconstruction at high strength (DLIR-H), over a 300 mm display field of view on a 512 × 512 matrix (in-plane pixel size 0.59 × 0.59 mm); synthetic monoenergetic images were generated from this reconstruction. Volume CT dose index was 4.57 mGy and dose-length product 168.57 mGy·cm for the helical acquisition (total examination dose-length product 178.64 mGy·cm). Sixty milliliters of iohexol 300 mg I/mL were injected intravenously at 3.5 mL/s, followed by a 40 mL saline flush at the same rate, corresponding to a total iodine load of 18 g. Acquisition was triggered visually by the operator on bolus-tracking monitoring images, followed by a diagnostic delay of 3.0 s.

Spectral post-processing was performed on a dedicated workstation (AW VolumeShare 7, GE HealthCare) from the spectral data set, using an iodine–water material decomposition basis pair for both the synthetic monoenergetic reconstructions and the virtual non-contrast images. Attenuation measurements were obtained on synthetic monoenergetic images at 55 keV, the energy level used for routine clinical reading of CT pulmonary angiography in our institution. A region of interest was drawn within the right main pulmonary artery filling defect on 3 consecutive slices while avoiding the vessel wall, and the reported value is the mean of these 3 measurements. Measurements were performed in consensus by a radiology resident with 2 years of experience and a board-certified radiologist with 15 years of experience.

Upon ICU admission, CTPA confirmed the presence of a large intraluminal filling defect in the right main pulmonary artery measuring 30 × 15 mm, as well as additional smaller filling defects in the segmental branches of the left lower lobe (Fig. 1A and B). The CT obstruction index was 35%. CT number measurements within the right main pulmonary artery filling defect demonstrated negative attenuation values, with a mean of −86.6 HU across 3 consecutive slices (range −92.7 to −83.4 HU; region of interest area approximately 32 mm^2^; mean within-ROI standard deviation 21.9 HU), consistent with fat. The segmental filling defects were too small for reliable region-of-interest measurement. The known right lower lobe pulmonary mass was stable compared to a prior CT performed 9 days earlier (Fig. 1C).

As the CT acquisition was performed using a dual-energy protocol, spectral analysis was performed on the workstation described above. Synthetic spectral CT number curves were generated for a single-slice region of interest placed within the fat embolus in the right main pulmonary artery and a reference region of interest placed in a paraspinal skeletal muscle. The spectral curves demonstrated divergent behavior at lower photon energies: the fat embolus exhibited increasingly negative CT number values at lower keV levels, reaching approximately −122 HU at 40 keV and −83 HU at 55 keV, and approximately −64 HU at 70 keV, while the soft tissue reference showed increasing values at low keV, reaching approximately +71 HU at 40 keV (Fig. 2A and C). A virtual noncontrast image generated from iodine vs water material decomposition supported the fatty nature of the filling defect, which remained clearly visible after removal of iodine-enhanced structures (Fig. 2B).

**Fig. 2:**
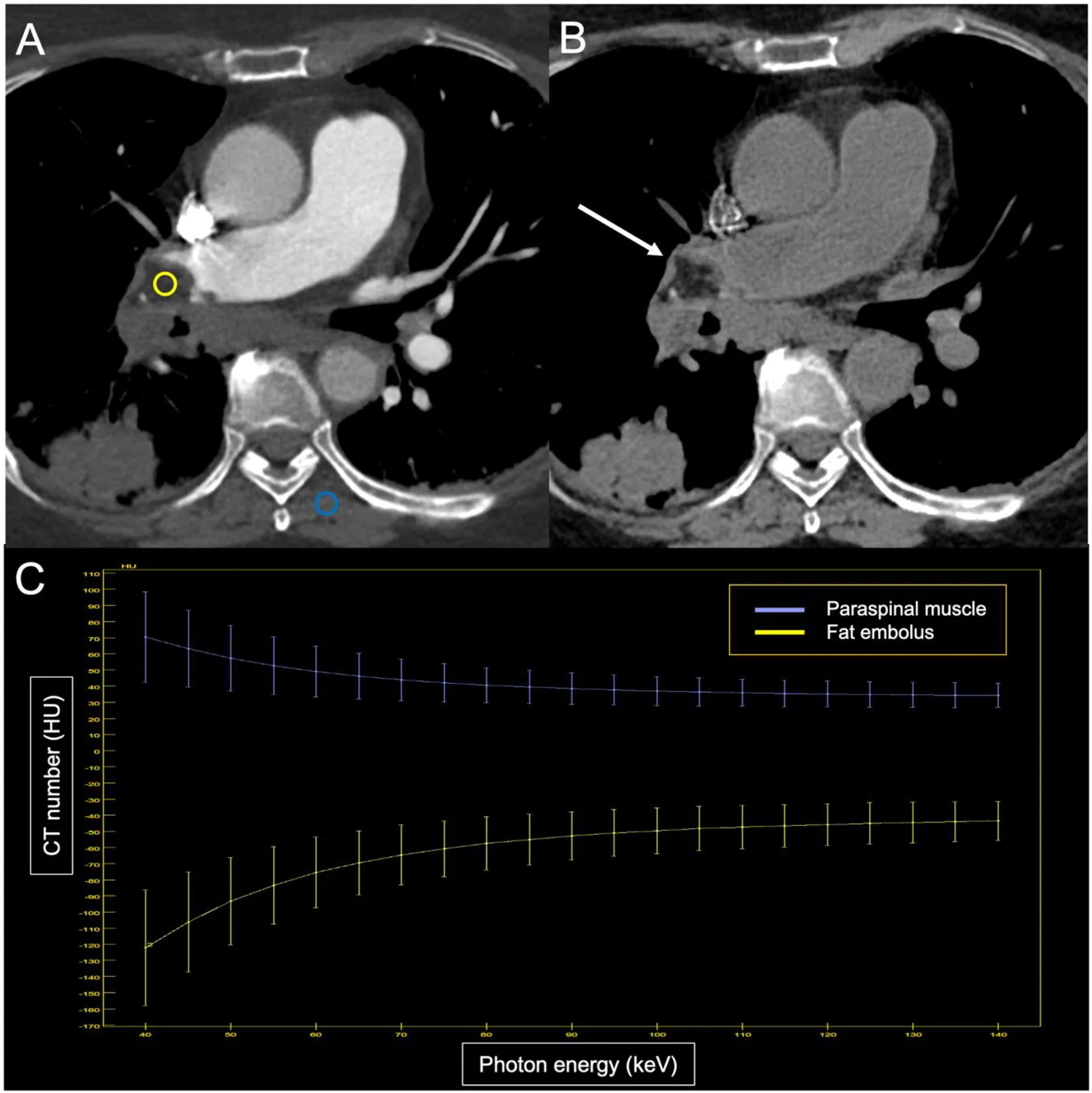
Dual-energy CT spectral analysis. (A) Axial pulmonary angiography (CTPA) showing the 2 regions of interest: 1 (yellow) placed within the fat embolus in the right main pulmonary artery and 1 (blue) placed in a paraspinal skeletal muscle. (B) Virtual noncontrast image (iodine vs water material decomposition) showing persistent visualization of the fat embolus after removal of iodine contribution, exhibiting a high contrast compared to the surrounding blood pool. (C) Synthetic spectral-based CT number curves demonstrate divergent behavior at low photon energies: the fat embolus (yellow curve) shows increasingly negative CT number values at lower keV, while the soft tissue reference (blue curve) shows increasing values at low keV due to the photoelectric effect and iodine enhancement.

The right-to-left ventricular diameter ratio (RV/LV) was less than 1, with no signs of right heart strain. Small bilateral pleural effusions with bibasal atelectasis were noted.

Given the absence of right heart strain and the hemodynamic improvement under vasopressor support, thromboaspiration was not indicated. A retrievable IVC filter (ALN) was placed on the same day for venous thromboembolism prophylaxis, via a right common femoral vein approach under fluoroscopic and ultrasound guidance, without immediate complication. Therapeutic anticoagulation with unfractionated heparin was subsequently initiated and progressively uptitrated, primarily to prevent secondary thrombotic complications in the postoperative oncological setting rather than to treat fat embolism itself.

The patient was successfully extubated 2 days postoperatively. Her ICU course was complicated by right basal atelectasis managed with noninvasive ventilation, microcytic anemia requiring red blood cell transfusion and intravenous iron supplementation, hypernatremia secondary to polyuria, and a nonanion gap metabolic acidosis with positive urinary anion gap, raising suspicion for renal tubular acidosis. Transthoracic echocardiography performed postoperatively confirmed preserved biventricular function with no right heart dilatation.

Following clinical improvement, the patient was transferred to a regional intensive care unit 5 days after the surgery for ongoing management. She was discharged with therapeutic anticoagulation maintained for the cumulative thrombotic risk (active malignancy, recent surgery, immobilization, indwelling IVC filter) in the absence of an independent indication such as atrial fibrillation or documented deep vein thrombosis, outpatient orthopedic follow-up, and a scheduled consultation for elective IVC filter retrieval at 5 months.

## Discussion

This case illustrates a rare and diagnostically challenging presentation of macroscopic pulmonary fat embolism occurring in the specific context of cemented hemiarthroplasty for a pathological femoral lesion secondary to bone metastases from NSCLC. Several features of this case merit discussion: the relationship between fat embolism syndrome and bone cement implantation syndrome, the pathophysiology of fat embolization in the oncological setting, the pivotal diagnostic role of CTPA with CT number measurements, the novel contribution of dual-energy CT spectral analysis, and the therapeutic challenges posed by this fragile patient population.

Terminology deserves clarification in this case. Fat embolism syndrome is a clinical diagnosis, usually based on the Gurd and Wilson criteria, which require one major criterion, 4 minor criteria and fat macroglobulinemia [1]. Our patient met one major criterion, respiratory failure requiring approximately 40 hours of mechanical ventilation, which was partly confounded by a concurrent community-acquired pneumonia. No petechial rash was documented. Neurological status could not be assessed under sedation, and the hypoactive confusional state observed after extubation was attributed to intensive care delirium. Only 2 minor features were present, a heart rate of 121 beats per minute in a patient with known sinus tachycardia and a 34% fall in hemoglobin, whereas fever, jaundice, oliguria and thrombocytopenia were absent; fat macroglobulinemia was not assessed. The criteria for fat embolism syndrome were therefore not met. What was documented was macroscopic pulmonary fat embolism on imaging, occurring in the setting of bone cement implantation syndrome, defined by hemodynamic collapse at the time of cement pressurization [3]. We regard bone cement implantation syndrome as the intraoperative clinical syndrome and macroscopic fat embolism as its radiological correlate, and the term fat embolism syndrome is used in this report only for the published entity.

### Pathophysiology and risk factors

Fat embolization following orthopedic surgery is classically attributed to the mechanical pressurization of the medullary canal during cement implantation, which forces fat globules, bone marrow debris, and cement particles into the venous circulation [4,9,10]. The same mechanism is thought to underlie bone cement implantation syndrome (BCIS), a clinical syndrome well described in the context of cemented arthroplasty and associated with intraoperative hypoxemia, hypotension, and cardiovascular collapse [3]. In the present case, the extent of fat embolization was likely amplified by the underlying pathological nature of the lesion. Lytic bone metastases disrupt the structural integrity of the medullary barrier, potentially increasing the volume of fat and marrow content released into the circulation during surgical manipulation and cement pressurization [10]. To our knowledge, macroscopic fat embolism complicating cemented arthroplasty specifically for metastatic bone disease has rarely been reported in the literature, making this case particularly noteworthy [2,5,9].

The patient’s additional risk factors, including advanced age, poor cardiorespiratory reserve, and pre-existing anemia, may have further contributed to her vulnerability to the hemodynamic and systemic consequences of fat embolization [1,4,10].

### Diagnostic role of CTPA and CT number measurements

The diagnosis of fat pulmonary embolism on CTPA relies on (1) direct visualization of intraluminal filling defects with negative Hounsfield unit values, reflecting the fat content of the embolic material [6,11]; however, macroscopic fat emboli are directly visualized in the pulmonary vessels in only a minority of cases, with one literature review reporting a detection rate of 25.4% (31/122 patients) among FES cases with available CTPA [12], and (2) less specific indirect CT findings such as ground-glass opacities, nodular opacities, septal thickening, consolidation, and pleural effusion.

In our case, the mean attenuation of the main filling defect was −86.6 HU at 55 keV, and the spectral curve showed approximately −64 HU at 70 keV, the energy level closest to a conventional acquisition; both values are well within the fat density range of −50 to −150 HU reported in the literature for fat emboli [6]. Although attenuation values showed some variability, this is most likely explained by partial volume averaging, beam hardening artifacts, and heterogeneity of the embolic material.

Of note, the largest filling defect in the right main pulmonary artery measured 30 × 15 mm. Direct CT visualization of macroscopic fat within the pulmonary vasculature is uncommon, being reported in only a minority of fat embolism cases with an available CTPA [6,12].

It is important to distinguish fat pulmonary embolism from other causes of intraluminal filling defects on CTPA, including thrombotic embolism, air embolism, cement embolism, and tumor embolism, as these entities differ in their pathophysiology, CT attenuation characteristics, and management [6,7,13]. In this regard, systematic measurement of CT numbers within any filling defect is a simple yet critical step that can significantly impact diagnostic accuracy in the postoperative setting.

### Spectral characterization using dual-energy CT

Spectral characterization of macroscopic fat pulmonary embolism using dual-energy CT has rarely been reported. Tang et al. [8] demonstrated the detection of pulmonary fat embolism with dual-energy CT in an experimental rabbit model. In a previously reported human case, Ota et al. [14] described a patient with fat embolism syndrome following a femoral trochanteric fracture in whom CTPA showed no intraluminal filling defect; dual-energy CT was used to generate iodine perfusion maps, which demonstrated peripheral perfusion defects consistent with microvascular occlusion by small fat particles [14]. Because no macroscopic embolus was visible in that case, no attenuation measurement, spectral attenuation curve or virtual non-contrast reconstruction of an embolus was performed. Our case differs from previous reports in that a spectral attenuation curve was obtained from a macroscopic fat embolus itself, paired with a soft-tissue reference region of interest and complemented by iodine–water virtual noncontrast imaging.

A recognized advantage of dual-energy CT acquisition for suspected PE is the ability to reconstruct virtual monoenergetic images at low keV levels, which have been shown to improve pulmonary artery opacification and diagnostic confidence in cases of suboptimal contrast enhancement, and may even salvage otherwise nondiagnostic examinations [15,16]. This is particularly relevant in hemodynamically unstable patients, in whom contrast bolus timing may be suboptimal.

In the present case, the dual-energy acquisition also enabled the generation of synthetic CT number curves demonstrating a characteristic divergence between the fat embolus and surrounding reference soft tissue at lower photon energies (Fig. 2C). The fat embolus showed progressively decreasing CT number values at lower keV levels, reflecting the absence of iodine uptake or hemoglobin and the inherent low attenuation of adipose tissue. In contrast, the soft tissue reference showed the expected increase in CT number at low keV, attributable to the higher probability of photoelectric effect and residual iodine enhancement. This divergent spectral behavior supports the fat composition of the embolus beyond conventional single-energy attenuation measurements.

Furthermore, the virtual noncontrast image generated through iodine vs. water material decomposition allowed clear visualization of the fat embolus by removing iodine-enhanced blood from the image, effectively isolating the nonenhancing fat component (Fig. 2B). These findings suggest that dual-energy CT with spectral analysis and material decomposition may serve as a valuable complementary tool for the characterization of intraluminal filling defects of uncertain nature. In particular, fat emboli would be expected to show decreasing attenuation at low keV and persistent low signal on virtual non-contrast maps, whereas acute thrombotic emboli, which may demonstrate some degree of iodine permeation, would show a different spectral signature and be less conspicuous on virtual noncontrast images [17]. This approach could prove especially useful in ambiguous cases where conventional attenuation measurements are inconclusive due to partial volume effects or mixed embolic material.

### Therapeutic considerations

The management of this patient was particularly challenging given the coexistence of multiple competing factors: the fresh surgical wound precluding early aggressive anticoagulation, the oncological context with palliative intent, the chronic kidney disease (KDIGO stage G4) limiting pharmacological options, and the hemodynamic fragility requiring prolonged vasopressor support.

Even though the CT obstruction index was 35% in a patient with co-morbidities [18], we found no signs of right heart strain, evidenced by an RV/LV ratio below 1 on both CTPA and TTE, suggesting a limited hemodynamic impact of the embolic burden. However, conventional risk stratification used for thrombotic pulmonary embolism may not be directly applicable in the context of fat embolism [19].

The treatment of fat embolism remains essentially supportive, and no specific pharmacological therapy has demonstrated consistent efficacy [10]. In a recent pooled analysis of 136 published cases of FES, only 5 patients (3.7%) received anticoagulant therapy, with respiratory supportive care being the mainstay of treatment in 93.4% of cases [20]. The role of anticoagulation in isolated fat embolism is controversial. Unlike thrombotic pulmonary embolism, routine anticoagulation is generally not recommended in pure fat embolism, as heparin does not dissolve fat emboli and may paradoxically potentiate FES pathogenesis by activating lipase and increasing circulating free fatty acid concentrations [1,21]. Furthermore, heparin carries a significant hemorrhagic risk in the postoperative setting [1,10]. Other agents including corticosteroids and dextran have been proposed but have shown limited efficacy and are still controversial [1,2,10]. This clinical dilemma is well illustrated in a reported case of FES coexisting with thrombotic pulmonary embolism, in which heparin was discontinued because the FES was judged more clinically severe, and only restarted once the syndrome had resolved [21].

In our case, the decision to initiate therapeutic anticoagulation with unfractionated heparin was motivated by the overall thrombotic risk profile, including postoperative immobilization, active malignancy (NSCLC stage IVb), and the presence of an IVC filter, rather than by the fat embolism itself. No independent indication for therapeutic anticoagulation, such as atrial fibrillation or documented deep vein thrombosis, was identified. Therapeutic anticoagulation was maintained at discharge for the same reasons, reflecting a pragmatic approach to the cumulative thrombotic risk in this complex oncological patient.

The retrievable IVC filter was placed for venous thromboembolism prophylaxis in a patient at high thrombotic risk—active metastatic malignancy, major orthopedic surgery and prolonged immobilization—during a period in which therapeutic anticoagulation could not yet be safely established. This decision was independent of the fat embolic event, and the filter was not placed to prevent further fat embolization. There are no guidelines for the use of IVC filters in fat embolism, and the evidence is limited to isolated case reports [22]. In one reported case, a large fat embolus was found in the inferior vena cava and iliac veins below an IVC filter after surgery for metastatic disease of the acetabulum and proximal femur, and was associated with renal transplant dysfunction [23]. A retrievable filter was selected in our patient with planned removal at 5 months, in accordance with current guidelines recommending retrieval once the thromboembolic risk has sufficiently decreased [24].

This report has limitations inherent to a single case, which preclude generalization. No histological confirmation of the fat nature of the embolic material was obtained, although the characteristic negative attenuation values and spectral signature provide consistent imaging evidence. Quantitative measurement was limited to the main filling defect, as the segmental defects were too small for reliable region-of-interest placement given partial volume averaging from the adjacent enhanced blood pool and vessel wall. Within the main defect, a relative standard deviation of approximately 25% suggests that some individual pixels may not have been within the fat attenuation range, even though the mean value remained within it. Long-term follow-up data regarding the resolution of the fat emboli were not available, as the patient was transferred to a regional hospital for ongoing care.

## Conclusion

Macroscopic pulmonary fat embolism is a potentially life-threatening complication of cemented arthroplasty in patients with lytic bone metastases, and may occur in the setting of bone cement implantation syndrome without fulfilling the established clinical criteria for fat embolism syndrome. Attenuation measurement within any intraluminal filling defect on CTPA, complemented where available by dual-energy spectral analysis, may allow differentiation from thrombotic embolism, although confirmation in larger series is required. In oncological patients at high thrombotic risk, the use of anticoagulation and IVC filters after such an event requires individualized multidisciplinary discussion.

## Ethics approval

Institutional ethics committee approval was not required for this single-patient case report, in accordance with local regulations.

## Declaration of generative AI and AI-assisted technologies in the writing process

The authors acknowledge the use of artificial intelligence-based language tools to assist with phrasing and editing of the manuscript. All scientific content, discussion, and conclusions are solely the responsibility of the authors.

## Patient consent

Written informed consent for publication of this case report and associated images was obtained from the patient.
